# Recurrent Bronchospasm During Intra-arterial Chemotherapy via IMAX Access for Retinoblastoma: A Case Report

**DOI:** 10.7759/cureus.105070

**Published:** 2026-03-11

**Authors:** Ruiyang Huang, Pragat Muthu, Maria Najera

**Affiliations:** 1 Anesthesia, University of Miami Miller School of Medicine, Miami, USA; 2 Ophthalmology, University of Miami Miller School of Medicine, Miami, USA; 3 Anesthesiology, Jackson Medical Center, Miami, USA

**Keywords:** airway management, anesthesiology, bronchospasm, intra-arterial chemotherapy, parasympathetic reflex, perioperative management, perioperative safety, retinoblastoma, trigeminocardiac reflex

## Abstract

Intra-arterial chemotherapy (IAC) has become an increasingly popular approach for treating advanced retinoblastoma. It offers targeted drug delivery and reduced systemic toxicity when compared to systemic chemotherapy. Respiratory complications are rare, but this case presents a patient who experienced recurrent episodes of intraoperative bronchospasm possibly linked to an anatomical variation. A six-month-old female with Group D retinoblastoma in her right eye underwent five cycles of IAC within six months of diagnosis. Due to failed visualization of the right ophthalmic artery during angiography, all infusions were delivered through the anastomosis of the internal maxillary artery (IMAX) with the ophthalmic circulation. The patient experienced intraoperative bronchospasm during the first, second, and fifth cycles, each time occurring after chemotherapy administration. Each episode presented with oxygen desaturation, increased airway pressures, and diminished breath sounds and was resolved with epinephrine and albuterol. No mechanical airway issues or systemic allergic responses were present, and cycles 3 and 4 proceeded without bronchospasms. Intraoperative bronchospasm during pediatric IAC has been rarely reported and remains poorly understood. This case adds to the body of literature describing severe and sometimes repeat, respiratory complications in IAC. Additionally, in our patient, drug delivery was always via an alternate route through the IMAX. The IMAX lies adjacent to branches of the trigeminal nerve path. We hypothesize that the pulsatile IAC administration near these sensory centers may have triggered a trigeminocardiac reflex, resulting in parasympathetic activation, bronchospasm, and bradycardia. This highlights a risk of IAC and the importance of perioperative preparedness when standard access is not possible during ophthalmic IAC, especially for patients with anatomic variations.

## Introduction

Retinoblastoma is one of the most common primary intraocular malignancies of childhood, especially among those diagnosed before the age of 5 [1,2]. In recent years, intra-arterial chemotherapy (IAC) has emerged as a targeted treatment strategy that delivers high concentrations of chemotherapeutic agents directly to the tumor [3-5]. The standard technique involves catheter entry through the femoral artery and chemotherapy delivery into the ophthalmic artery via the internal carotid artery (ICA) [6]. This approach minimizes systemic toxicity while maximizing local efficacy.

While IAC is generally well tolerated, its procedural complexity and intra-arterial drug delivery carry inherent risks. Reported complications are most commonly local, including periocular edema, ptosis, vitreous hemorrhage, and retinal detachment [3,7,8]. Systemic adverse effects are more rare due to the localized nature of drug administration, and intraoperative respiratory events such as bronchospasm are uncommon, seen at a rate of 5.8% [3].

Here, we describe the case of a pediatric patient undergoing IAC for unilateral retinoblastoma in the right eye who developed intraoperative bronchospasm during the first, second, and fifth rounds of the planned treatments. Each episode occurred directly after administration of the chemotherapy. Each bronchospasm required pharmacologic intervention with epinephrine and resolved promptly, raising important questions about the mechanism of this unexpected complication. Although rare, similar respiratory complications during IAC have been reported; however, the etiology remains poorly understood and recurrent episodes are unusual [9-12]. Also notably, this patient required drug delivery via the internal maxillary artery (IMAX) due to the absence of ophthalmic artery visualization, and to our knowledge, this is the first described case of recurrent bronchospasm during IAC performed exclusively through this alternative route. This case highlights the need for preparedness when managing these procedures under general anesthesia and adds to the literature describing this phenomenon.

## Case presentation

**Figure 1:**
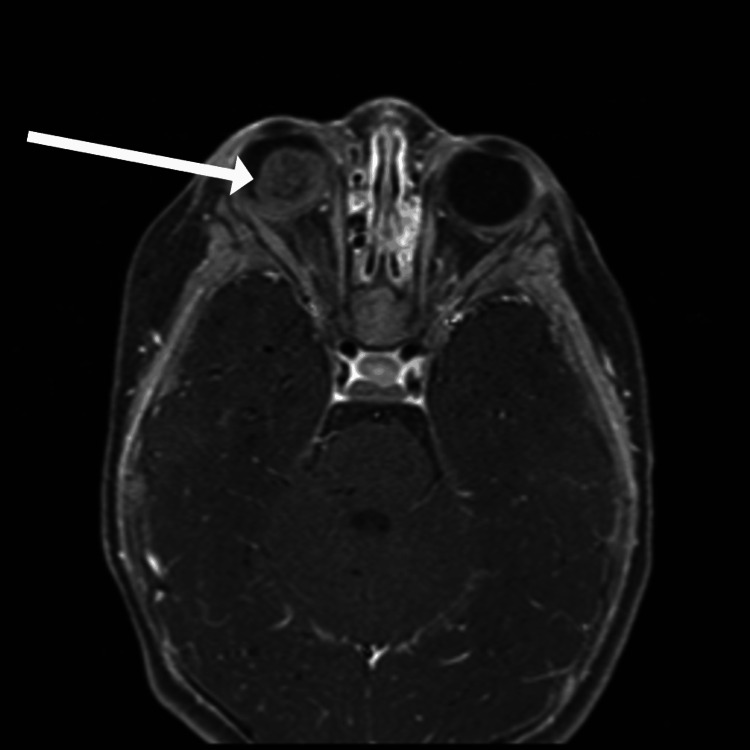
Axial view of MRI of the orbits during initial hospital stay. White arrow indicates intraocular mass within the right globe with internal restricted diffusion and microhemorrhage, consistent with retinoblastoma.

A six-month-old, 8.6 kg female, born at 36 weeks gestation via cesarean section due to placenta previa, presented with leukocoria in the right eye. Her mother initially noticed an abnormal brightness in her daughter's eye at three months of age, followed by a white pupillary reflex at four months. An MRI of the brain and orbits under sedation with propofol during the initial hospital stay revealed a 13 × 14 × 13 mm intraocular mass within the right globe with internal restricted diffusion and microhemorrhage, consistent with retinoblastoma. The lesion involved the inferior and posterior aspects of the globe and extended to the lens. The patient was diagnosed with unilateral, Group D retinoblastoma. The MRI of the brain and orbits showing the intraocular mass is shown in Figure 1.

She had no past medical or surgical history. Her family history was notable for multiple malignancies, including leukemia, brain cancer, oral cancer, and a sibling with autosomal recessive polycystic kidney disease. She lived with her parents and three siblings (ages 4, 7, and 11) and had no known allergies.

Due to scheduling delays for IAC, she received a single cycle of bridging intravenous carboplatin during this first hospital stay. She then underwent five IAC cycles, monthly. All procedures were performed under general anesthesia, with intra-arterial chemotherapy administered via the right IMAX using an anastomotic connection to the ophthalmic artery.

Technical approach and angiography

**Figure 2:**
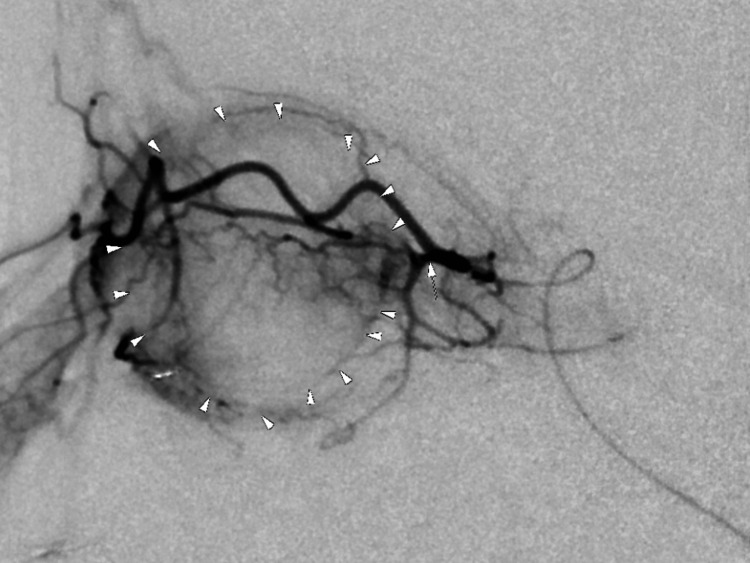
Digital subtraction angiography demonstrating choroidal blush following contrast delivery via the right IMAX. Arrowheads were added during image interpretation to highlight the area of choroidal perfusion, confirming successful anastomotic flow to the ophthalmic circulation in the absence of a directly visualized ophthalmic artery. IMAX: internal maxillary artery.

Across all five cycles, angiographic evaluation consistently failed to visualize the right ophthalmic artery via the internal carotid system. This necessitated selective catheterization of the right IMAX through the external carotid artery (ECA), which demonstrated a stable and favorable anastomosis with the ophthalmic artery. The IMAX consistently provided excellent choroidal blush, confirming successful delivery of intra-arterial chemotherapy. No catheter manipulation issues or vascular complications were reported. Digital subtraction angiography imaging demonstrating the successful delivery of the drug into the ophthalmic circulation is shown in Figure 2.

Endotracheal intubation was performed uneventfully for each cycle, and the airway was confirmed to be patent and well positioned. There were no signs of secretions or kinking, and decreased breath sounds were the primary auscultatory finding during each bronchospastic episode. No premedications were given during any of the cycles.

Cycle 1 

Induction and Anesthetic Course

The patient was given midazolam (0.5 mg) before arrival to the operating room. Induction was achieved with fentanyl (10 mcg), and propofol (15 mcg). The patient was intubated without complications and maintained on sevoflurane. After establishing adequate depth of anesthesia, a 22-gauge IV was placed in the left hand. Rocuronium (8 mg) was used for relaxation, and the patient was intubated with a Mac size 1 laryngoscope and a 3.5 mm endotracheal tube with a grade 1 view. 

Intraoperative Course

A catheter was introduced and advanced through the right internal carotid artery, and upon contrast administration, it was discovered that the right ophthalmic artery could not be visualized. The catheter was then withdrawn to the common carotid and advanced to the external carotid artery and passed to the IMAX. Contrast injected in the IMAX revealed a robust anastomosis to the right ophthalmic artery. Carboplatin (30 mg) and topotecan (0.3 mg) were injected in a pulsatile fashion over 30 minutes. Of note, during administration, the patient's heart rate decreased from the 140 beats per minute (bpm) range to 116 bpm. Immediately following administration of the chemotherapeutic agents, O_2_ saturation dropped to 75% from 100%. Mean arterial pressure (MAP) dropped from 44 to 33 mmHg. Tidal volume dropped from 56 mL to 6 mL, and peak inspiratory pressure rose from 11 cmH_2_O to 28 cmH_2_O. End-tidal CO_2_ (ETCO_2_) increased as well from 36 mmHg to 52.9 mmHg. 180 mcg of inhaled albuterol and 1 mcg of IV epinephrine drawn from a 1:1,000 (1 mg/mL) solution were administered when the desaturation first occurred. O_2_ saturation rose gradually to 90% within five minutes and another 1 mcg of epinephrine was given. The patient then began saturating at 100% O_2_ and was extubated successfully with no other complications. These intraoperative events are summarized in Table 1. 

**Table 1:** Timeline of intraoperative events during Cycle 1. IMAX: internal maxillary artery.

Intraoperative event timeline							
8:21 AM	8:24 AM	10:03 AM	10:40 AM	10:43 AM	10:43 AM	10:50 AM	10:55 AM
Induction started	Intubation complete	Successful access and visualization of the IMAX artery. Initiation of chemotherapy	Approximate end to 30 minute pulsatile infusion of chemotherapy	Bronchospasm, desaturation to 75%	Epinephrine and albuterol given	Patient O_2_ saturation returns to 100%	Awake extubation is complete

Reversal and Extubation

At the end of the three-hour procedure, the patient was given 32 mg of sugammadex (or 4 mg/kg she weighed 8 kg). She was extubated while awake with spontaneous breaths of tidal volume 28 mL. 

Postoperative Course

The patient was transferred directly to the pediatric intensive care unit (PICU) for 24-hour observation. She was hemodynamically stable and had an O_2_ saturation of 100% when leaving the operating room. She was observed for 24 hours post-procedure without complications and discharged home.

Cycle 2

Induction and Anesthetic Course

Induction was achieved with lidocaine (10 mg, 2%), fentanyl (10 mcg), and propofol (30 mcg). The patient was intubated without complications and maintained on sevoflurane. After establishing adequate depth of anesthesia, a 24-gauge IV was placed in the left hand. Rocuronium (8 mg) was used for relaxation, and the patient was intubated with a Miller size 1 laryngoscope and a 3.5 mm endotracheal tube with a grade 1 view. 

Intraoperative Course

Once again, following chemotherapy, the patient experienced O_2_ desaturation, this time from 100% to 86%. Tidal volume during the event was unrecorded, but peak inspiratory pressure rose from 19 cmH_2_O to 38 cmH_2_O. The heart rate fell from 143 bpm to 88 bpm. 1 mcg of IV epinephrine drawn from drawn from a 1:1,000 (1 mg/mL) solution was administered, along with 900 mcg of inhaled albuterol, and the patient’s O_2_ saturation rose to 86% after two minutes and was 100% seven minutes later. The MAP remained stable throughout the event this time at 49-50 mmHg and increased after epinephrine administration as expected. The bronchospasm event was during deep extubation after chemotherapy injection. These intraoperative events are summarized in Table 2. 

**Table 2:** Timeline of intraoperative events during Cycle 2. IMAX: internal maxillary artery.

Intraoperative event timeline							
8:09 AM	8:14 AM	9:14 AM	9:45 AM	10:18 AM	10:19-10:20 AM	10:22 AM	10:35 AM
Induction started	Intubation complete	Successful access and visualization of the IMAX artery. Initiation of Chemotherapy	Approximate end to 30 minute pulsatile infusion of chemotherapy	Bronchospasm, desaturation to 86%	Epinephrine (3 mcg/0.15 mL) given. Albuterol (900 mcg inhaled) given	Return to baseline oxygen saturation	Deep extubation complete

Reversal and Extubation

At the end of the 2.5-hour procedure, deep extubation was attempted. The patient experienced bronchospasm during this period and was unable to be ventilated. After administration of intervention, ventilation resumed, and the patient began breathing spontaneously.

Postoperative Course

The patient was discharged to the inpatient floor for observation. It was reported that there was difficulty finding lower extremity pulses postoperatively, and the lower extremities were cold. She was observed for four hours with adequate return of temperature and pulses to the lower extremities. Given the risk and concerns for thrombosis, a doppler US was done that showed adequate blood flow to both extremities. The patient was observed overnight and had an adequate pulse and temperature when discharged in the morning.

Cycle 3 

The third IAC cycle was completed without any respiratory or hemodynamic complications. The patient was induced with fentanyl (25 mcg), propofol (20 mcg), and maintained on sevoflurane. Rocuronium (10 mg) was used for neuromuscular blocking. Chemotherapy was administered with the same dose and in the same artery as the previous two cases. Care and attentive observation by the anesthesia team was planned during the pulsatile chemotherapy injections, with epinephrine and airway support immediately available should another event occur. The patient tolerated the procedure well. MRI performed post-procedure showed tumor regression from 13 × 14 × 13 mm to 8 x 9 x 7 mm and decreased vascularity. The patient was discharged on the day of the surgery.

Cycle 4

The fourth IAC cycle was also completed without any respiratory or hemodynamic complications. Induction was with fentanyl (10 mcg), propofol (30 mcg), and maintained on sevoflurane. Rocuronium (10 mg) was used for neuromuscular blockade. Chemotherapy was administered in the same fashion. Once again, careful attention especially during the chemotherapy administration was implemented. The patient tolerated the procedure well and was discharged the following day.

Cycle 5

Induction and Anesthetic Course

The fifth IAC cycle was induced and maintained with sevoflurane. Rocuronium (10 mg) was used for neuromuscular blockade. After establishing adequate depth of anesthesia, a 22 gauge IV was placed in the right antecubital fossa. The patient was intubated with a Miller size 1 Blade and 3.5 mm cuffed endotracheal tube with a grade 1 view. 

Intraoperative Course

Chemotherapy was administered in the same fashion. During the last 10 minutes of administration, the heart rate decreased from 143 to 124. Immediately after administration, O2 saturation decreased from 100% to 93%. Tidal volume also decreased from 60 mL to 39 mL. 3 mcg of IV epinephrine drawn from a 1:1,000 (1 mg/mL) solution was administered immediately following desaturation. Peak inspiratory pressure showed a slight increase from 17 cmH_2_O to 19 cmH_2_O, and ETCO_2_ and MAP remained stable. These intraoperative events are summarized in Table 3.

**Table 3:** Timeline of intraoperative events during Cycle 5. IMAX: internal maxillary artery.

Intraoperative event timeline							
8:20 AM	8:43 AM	9:42 AM	9:46 AM	9:47 AM	9:50 AM	10:10 AM	11:11 AM
Induction started	Intubation complete	Successful access and visualization of the IMAX artery. Initiation of chemotherapy	Desaturation to 93%	Epinephrine (1 mcg) given	Return to baseline oxygen saturation	Approximate end to 30 minute pulsatile infusion of chemotherapy	Successful awake extubation

Reversal and Extubation

At the end of the three-hour procedure, the patient was given 40 mg of sugammadex (or 4 mg/kg she weighed 9 kg). She was extubated while awake with spontaneous breaths of tidal volume 59 mL. 

Postoperative Course

Due to the previous history of complications postoperatively, the patient was kept overnight for observation. There were no acute events overnight, and the patient was discharged on postoperative day 1. 

Current Status

As of the fifth IAC cycle, the patient is awaiting follow-up evaluation by the ophthalmology team to assess treatment response. A determination will be made within one month regarding the necessity of additional IAC sessions. Should further treatment be required, the anesthesia team will maintain heightened vigilance during intra-arterial drug administration given the patient's prior bronchospastic episodes. Imaging has demonstrated therapeutic effects of treatment on decreasing tumor size and vascularity from 13 x 14 x 13 mm to 8 x 7 x 6 mm after the most recent cycle.

## Discussion

This case describes an uncommon and recurrent intraoperative bronchospasm event during IAC for unilateral retinoblastoma in an infant, coinciding with selective drug infusion via the right IMAX. The bronchospasm was observed during the first, second, and fifth IAC sessions, each time coinciding with intra-arterial administration of chemotherapy agents. Most reported instances of intraoperative bronchospasm in pediatric patients are isolated, and recurrent bronchospasm during repeated IAC procedures remains poorly understood and infrequently reported. This consistent recurrence suggests a specific, underlying physiologic trigger rather than an incidental or procedural variability. Understanding why this patient experienced this reaction repeatedly is critical for anticipating risk, refining procedural protocols, and improving perioperative safety in future patients undergoing this increasingly effective and popular treatment option for retinoblastoma. 

The most common causes of intraoperative bronchospasm, including anaphylactic reactions or airway manipulation, were first considered [13]. Although each episode was self-limited following administration of epinephrine and albuterol, the patient did not exhibit signs of a systemic allergic response such as rash, hypotension, or angioedema. Furthermore, mechanical issues such as endotracheal tube displacement, mucus plugging, or ventilator malfunction were not evident. In all cases, end-tidal CO₂ and tidal volume dropped precipitously, and airway pressures increased, consistent with acute bronchoconstriction. However, the consistent temporal relationship between drug infusion and the onset of symptoms raises the possibility of a reflexogenic component.

Similar cases of severe respiratory reactions during IAC procedures have been investigated in single-center studies by Kato et al. and Philips et al., both noting the incidence of significant respiratory complications in pediatric patients during this procedure [9,10]. While these reports recognize the increased need for vigilance in these procedures, the cause of the events remains not fully understood, and they call for more investigation into this physiologic reaction [9-12]. The specific association with right IMAX access described in our patient may offer more insight into a possible anatomic cause. 

Anatomically, this patient exhibited a notable vascular variant as the right ophthalmic artery could not be visualized via internal carotid artery (ICA) angiograms in any of the procedures. As a result, intra-arterial chemotherapy was delivered via the IMAX, a distal branch of the external carotid artery (ECA), which demonstrated robust anastomosis with the ophthalmic circulation. This route, while anatomically feasible, is not standard and introduces new neurovascular considerations.

The IMAX lies in close proximity to branches of the maxillary division of the trigeminal nerve within the pterygopalatine fossa. Stimulation of afferent sensory nerve endings in this area is known to provoke different parasympathetic reflexes such as the trigeminocardiac reflex (TCR), a brainstem reflex arc that involves trigeminal input and vagal efferent output [14,15]. The result of this reflex is increased parasympathetic output, which can cause bradycardia, hypotension, and in some cases, apnea and bronchoconstriction [14]. The mechanism is thought to involve parasympathetic activation through connections within the brain, which can trigger bronchial smooth muscle constriction through vagal innervation [16].

The TCR is most commonly seen in craniofacial and skull-base surgeries; however, the anatomic proximity of the IMAX to branches of the maxillary division of the trigeminal nerve raises the possibility of a similar parasympathetic reflex being triggered during the pulsatile injection of the chemotherapeutic agents [17-19]. Mechanical or chemical stimulation of the nerve may have provoked a similar parasympathetic response, which led to bronchoconstriction and bronchospasm [14]. In our patient, transient bradycardia was observed concurrently with each bronchospastic episode, further supporting a parasympathetic reflex mechanism. If the cause of the bronchospasm was a reaction to chemotherapy or another drug, tachycardia would be expected during anaphylaxis. Additionally, our patient was exposed to chemotherapy and anesthetic drugs in other settings, such as during her first dose of intravenous bridging therapy and during sedation and anesthesia for MRI imaging and exams under anesthesia. Neither of these scenarios caused allergic or anaphylactic reactions during intravenous drug administration.

Understanding this potential mechanism of recurrent bronchospasm in this patient has significant implications. First, it highlights the importance of recognizing variant vascular anatomy during ophthalmic IAC, as catheterization through the ECA/IMAX may carry unique risks not present with standard ICA-based infusion. Second, it underscores the need for heightened vigilance and preparedness during such procedures for the perioperative team. In our case, anesthesia providers were prepared for repeat events after the second occurrence. An anesthesia attending was in the operating room during the chemotherapy injection, and epinephrine was available for immediate administration. In the fifth round of chemotherapy, the bronchospasm and resulting hypoxia were objectively milder, with a less severe drop in oxygen saturation from 100% to 93%. This attenuated response may be partially attributed to the quicker response time of the anesthesia team in recognizing the event. Third, it raises questions about possible pre-treatment or adjustments in anesthetic depth. Some studies have reported the use of pretreatment with anticholinergics or inhaled albuterol for the prevention of bronchospasm in IAC, but the efficacy of these interventions remains uncertain [5,20]. 

This case contributes to the growing recognition of neurovascular reflexes during interventional procedures and suggests that variant anatomy may expose patients to unexpected autonomic responses. It adds to the small body of literature describing severe respiratory complications and bronchospasm during IAC. While definitive confirmation of a parasympathetic reflex arc in this context is limited by the lack of real-time neurologic or vagal monitoring, the consistent clinical pattern supports this as a possible mechanism. Future studies should further characterize the neurovascular landscape of pediatric IAC and explore strategies to anticipate and mitigate reflexogenic complications.

## Conclusions

This case illustrates an uncommon, reproducible intraoperative complication in a pediatric patient undergoing IAC. It raises the possibility of a reflexogenic mechanism linked to variant vascular anatomy with IMAX access. While the exact mechanism remains speculative, the findings underscore the importance of careful preprocedural planning, awareness of anatomic variants, and intraoperative vigilance for timely intervention. Further investigation is warranted to better understand reflexogenic responses in similar interventional contexts and to establish preventative strategies for high-risk patients.
